# Dynamic Modeling and Interactive Performance of PARM: A Parallel Upper-Limb Rehabilitation Robot Using Impedance Control for Patients after Stroke

**DOI:** 10.1155/2018/8647591

**Published:** 2018-04-05

**Authors:** Hui Guang, Linhong Ji, Yingying Shi, Berno J. E. Misgeld

**Affiliations:** ^1^Department of Mechanical Engineering, Tsinghua University, Beijing, China; ^2^Department of Mechanical Engineering, Beihang University, Beijing, China; ^3^Helmholtz-Institute for Biomedical Engineering, RWTH Aachen University, Aachen, Germany

## Abstract

The robot-assisted therapy has been demonstrated to be effective in the improvements of limb function and even activities of daily living for patients after stroke. This paper presents an interactive upper-limb rehabilitation robot with a parallel mechanism and an isometric screen embedded in the platform to display trajectories. In the dynamic modeling for impedance control, the effects of friction and inertia are reduced by introducing the principle of virtual work and derivative of Jacobian matrix. To achieve the assist-as-needed impedance control for arbitrary trajectories, the strategy based on orthogonal deviations is proposed. Simulations and experiments were performed to validate the dynamic modeling and impedance control. Besides, to investigate the influence of the impedance in practice, a subject participated in experiments and performed two types of movements with the robot, that is, rectilinear and circular movements, under four conditions, that is, with/without resistance or impedance, respectively. The results showed that the impedance and resistance affected both mean absolute error and standard deviation of movements and also demonstrated the significant differences between movements with/without impedance and resistance (*p* < 0.001). Furthermore, the error patterns were discussed, which suggested that the impedance environment was capable of alleviating movement deviations by compensating the synergetic inadequacy between the shoulder and elbow joints.

## 1. Introduction

Stroke is caused by cerebrovascular accident and is one of the leading diseases of disability, motor disorder, and deterioration of activities of daily living (ADL). The incidences in the European Union and the United States are approximately one million and 0.8 million per year, respectively [1, 2], and thirty percent of patients suffer recurrent attacks, which results in increasing demand for rehabilitation services.

For patients after stroke, the task-repetitive training has been demonstrated to be effective in improving their upper and lower extremity functions and ADL [3]. To meet the requirement for repetitive training, various upper-limb rehabilitation robots have been developed over the past twenty years, which are generally classified into two categories [4]: end-effector robots, such as DIAGNOBOT [5], CARR [6], MIT-MANUS [7], MIME [8], GENTLE/s [9], and exoskeleton robots, such as CADEN-7 [10], RUPERT [11], BONES [12], and ARMin [13]. Since the robotic rehabilitation exhibits the advantages in terms of high-dosage, high-intensity, and task-specific training [14], randomized controlled trials comparing the robot-assisted and conventional therapy have yielded significant effects of robots on the improvements of limb function [15, 16] and even ADL [17].

Although many robots for the upper-limb rehabilitation have been developed, mechanical design, control, and training methods remain an area of interest. As pointed out by Belda-Lois et al. [18], robot-assisted rehabilitation could be enhanced by means of precisely controllable assistance or resistance, enhanced training motivation through interactive feedback, and quantifiable and objective measures of subject performance. Besides, cost should also be considered [19].

Generally, the exoskeleton robots take individual joint motions into account to minimize abnormal postures and joint motions. Nevertheless, due to the complexity of the human upper-limb anatomy, the instantaneous centers of rotation of the upper-limb joints are changed with movement [20], which causes the inconvenience of joint axis alignments and raises interactive force between human and robots [21], thereby obstructing the development and application of the exoskeleton robots. In contrast to exoskeleton robots, end-effector robots are simple and cost-effective and can adapt to patients with diverse somatotypes [4]. Despite the disadvantage of end-effector robots in joint training, extensive research has also demonstrated their effectiveness and superiority for improving upper-limb function and ADL in comparison with conventional therapies [14].

Compared to serial mechanisms, parallel mechanisms exhibit inherent advantages of low inertia, high stiffness, and satisfactory payload capability [22, 23]. More importantly, as the end-effector is controlled in parallel, the errors of the joint control are not accumulated and amplified by serial counterparts, and thus the manipulator is less affected by joint clearance and has higher precision in aspects of position, stiffness, and interactive force control [12]. Therefore, parallel manipulators have been recently applied to rehabilitation robots, including shoulder [12, 24], wrist [25], hip [26], and upper-limb rehabilitation devices [27].

Another issue is that understanding sensorimotor physiology is more imperative prior to developing a rehabilitation robot. For instance, one aspect is how individual joints, as well as segments, are coordinated to achieve the task. In physiology, limb movements are perceived in an egocentric reference frame, in which targets are defined with respect to the trunk or head. In contrast, an allocentric reference frame represents the coordinate system external to the body [28]. However, for current training robots, target and actual trajectories are presented in a standing monitor, which is a virtual environment based on the allocentric reference frame for patients. Thus, patients are required to transform the targets and movements in the virtual environment to the egocentric reference frame to accomplish the task, causing difficulties in perception and sensorimotor control. Besides, it might weaken the effect of proprioceptive training since the actual positions do not directly correspond to virtual positions.

Based on the issues discussed above, a novel end-effector-based upper-limb rehabilitation robot, which is named PARM, is developed with a parallel mechanism and patient-frame-based interactive feedback to enhance training performance. Distinct from other rehabilitation robots, a monitor was embedded in the platform to show target and actual trajectories, providing isometric direct visual feedback for patients. The trajectories displayed on the platform screen were the same as the actual trajectories in movement space, particularly in the aspects of scale, position, and direction. Therefore, patients could perceive targets and movements in the egocentric reference frame, which should improve the motor recovery and proprioceptive training. As the precise control of position, stiffness, and force contributes to training effects [18], the impacts of friction and acceleration were incorporated to improve the control precision. Consistent with the robots such as MIT-MANUS [7], an assist-as-needed strategy was also introduced in PARM to improve interaction between patients and robots. In the assist-as-needed control, patients determine the manipulator in terms of position, velocity, and acceleration; thus, the reference positions are variable with the movement and associated with real-time deviations.

To summarize, the novelty of our work is the strategy based on the orthogonal deviation for assist-as-needed impedance control, which aims to obtain the equilibrium positions and calculate impedance force, and the hardware which adopts parallel mechanism and isometric visual feedback. Simulated and experimental results validated the dynamic modeling and impedance control. Since the mechanism of the impedance control contributing to the motor coordination is still less clear, the functional interaction between impedance control and movements was also discussed.

## 2. Apparatus and Specification

**Figure 1:**
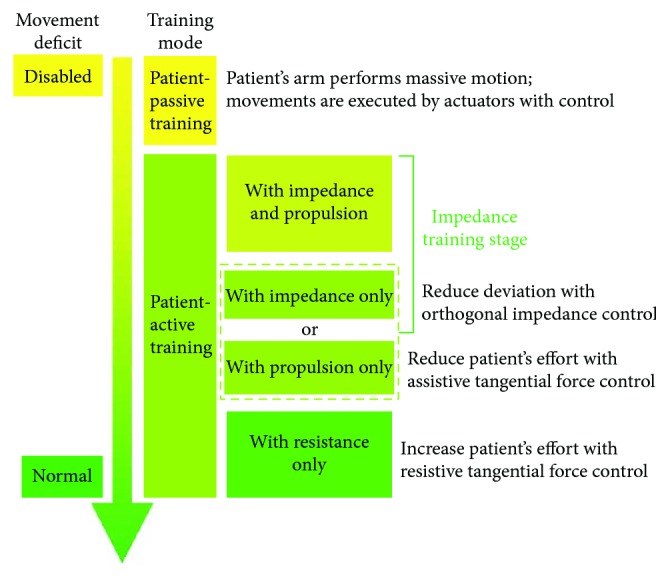
Training modes of PARM for stroke patients.

The rehabilitation robot PARM aims to improve the motor performance of stroke patients by enhancing movement interaction between the patients and the robot. This interactive robot incorporates multiple training modes for patients with diverse disability and recovery stages, which are summarized as patient-passive training and patient-active training (Figure 1). Arbitrary reference trajectories are predefined by therapists prior to training. In the patient-passive training, the movements are entirely actuated by the robot with position control, in which the robot is a mechanical admittance whereas the patient's arm is regarded as an impedance. Contrastively, in the patient-active training, movements are initiated and actuated by patients with partially assistance or resistance. For instance, in the training with impedance and propulsion, the impedance force towards the target trajectory aims to rectify deviations, while the propulsive force towards the movement direction could reduce the active force of the patient, which decreases the task difficulty. Conversely, the resistance force against the movement direction increases the movement effort.

To increase the benefit of robot-aided therapy, control schemes should be customized for individuals and adopted to patients' deficits in upper-limb motor function, based on their poststroke stages and clinical assessments. For subacute patients, since they are generally unable to perform voluntary arm-reaching tasks due to dystonia, training is mainly executed in patient-passive modality. For chronic patients (more than 6 months poststroke), robot-aided therapies are generally performed in patient-active modalities to enhance patient engagement. Specifically, when patients could perform inaccurate arm-reaching tasks, impedance control is applied for this training stage to rectify deviations and improve their abilities in motor control. Besides, for patients with low strength, propulsion in movement direction is included to reduce the movement effort. However, for the patients having coordinated motor control, impedance control is removed. Instead, resistance in movement direction might be involved to match their motor function and improve training outcomes.

**Figure 2:**
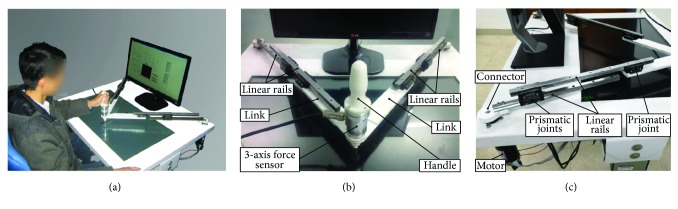
Mechanical construction of PARM. (a) Robot overview. (b) Robot parallel mechanism. (c) Motor and joint connections.

PARM mainly consisted of a lifting platform, two monitors, a five-bar parallel mechanism with two motors and actuators, and a three-axis force sensor (Figure 2). A horizontal monitor was embedded in the platform to display the reference trajectory and actual trajectories, providing direct visual feedback for patients, while a standing monitor was used to display the configuration of training parameters and quantitative assessments. Additionally, the inclination and height of the platform could be adjusted to make the trajectories conveniently observed for patients.

The five-bar parallel mechanism RPRPR (revolute-prismatic-revolute-prismatic-revolute) is shown in Figures 2(b) and 2(c). Linear rails were adopted to increase movement range of end-effector and improve kinematic precision, and linear bearing blocks constituted the prismatic joints to reduce friction. Each side consisted of three prismatic joints and two linear rails, and thereby the minimal length of each side was the length of a rail, while the maximal length was the sum of two rails and a link. During movement, the length of two sides was accordingly adjusted to the two revolute joints controlled by the motors.

The parallel mechanism was actuated by two Maxon RE50 DC motors with shaft keys, and connectors concatenated the linear rails and motors by screws and shaft keys, respectively. The motors were fixed on the platform and in serial with angle encoders, and the nominal voltage, maximum torque, and torque constant of the motor were 36 V, 418 mNm, and 60.4 mNm/A, respectively. The Maxon gearboxes EP52C, whose gear ratios were 43 : 1, modulated the motor outputs. The motors were actuated by Maxon EPOS2 70/10, and control programs were coded in LabVIEW (NI, USA).

**Figure 3:**
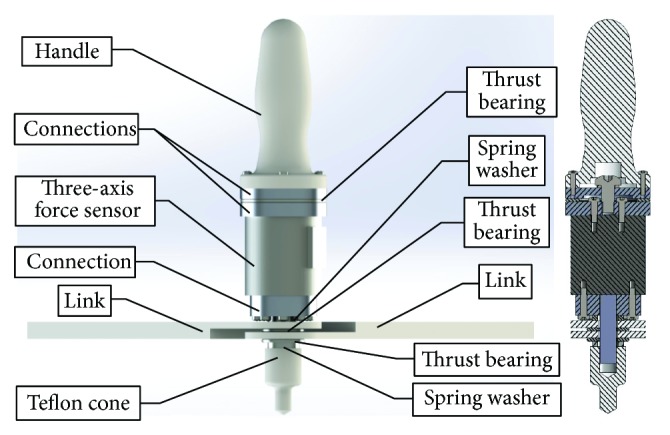
End-effector components.

In the patient-active training, an assisted-as-needed strategy was introduced by employing impedance control. To improve the control precision of manipulator impedance, a three-dimensional force sensor was mounted on the end-effector, which could additionally record the interactive force between patients and the robot for quantitative assessments. The sensor signals were collected by Arduino board (Mega 2560) and subsequently transmitted to PC through USB serial communication. The end-effector components are shown in Figure 3. Since the angle between the upper-limb and the links changed with movements when patients hold the handle, the handle had one independently rotational degree of freedom (DoF) with respect to the links, and the revolute friction was reduced by thrust bearings. The cone below the end-effector was used to indicate movement positions, and Teflon was adopted to reduce the friction with screen. In addition, the revolute joint of two links was constituted by an axis, and friction was also reduced by thrust bearings.

## 3. Kinematic and Dynamic Modeling

### 3.1. Kinematics of PARM

**Figure 4:**
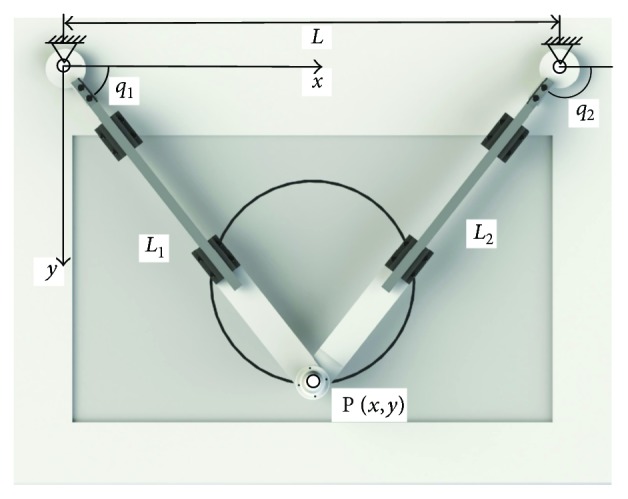
Kinematic diagram of PARM.

PARM has two DoF actuated by two servo motors, and the kinematic diagram is shown in Figure 4. The end-effector position *P*(*x*, *y*) was determined by the joints *Q*(*q*_1_, *q*_2_), which is given by

(1) $$\begin{array}{c} x=\frac{L\tan q_{2}}{\tan q_{2}-\tan q_{1}},\\ y=\frac{L\tan q_{1}\tan q_{2}}{\tan q_{2}-\tan q_{1}}, \end{array}$$

where *L* means the distance between two joints.

Patient-passive control is based on the inverse kinematics of the robot arm. For continuous predefined trajectories *g*(*x*, *y*, *t*), controlled joints *Q* is calculated as

(2) $$\begin{array}{c} q_{1}=\tan\frac{y}{x},\\ q_{2}=\pi -\arctan\frac{y}{L-x}. \end{array}$$

The calculated joint angles are implemented with position control of servo motors.

Deriving (1), Jacobian matrix **J**_**p**_ denotes the relationship between the end-effector and joint velocity, which is given by

(3) $$\begin{array}{c} \left[\begin{array}{l} \dot{x}\\ \dot{y} \end{array}\right]=J_{p}\left[\begin{array}{l} {\dot{q}}_{1}\\ {\dot{q}}_{2} \end{array}\right]. \end{array}$$

Besides, link lengths *L*_1_ and *L*_2_ were adjusted automatically to the joint angles *q*_1_ and *q*_2_ as

(4) $$\begin{array}{c} L_{1}=\frac{L\sin q_{2}}{\sin q_{2}-q_{1}},\\ L_{2}=\frac{L\sin q_{1}}{\sin q_{2}-q_{1}}. \end{array}$$

Similarly, the relation between the elongation velocity of the two links $\dot{L}\left({\dot{L}}_{1},{\dot{L}}_{2}\right)$ and the joint angular velocity $\dot{Q}\left({\dot{q}}_{1},{\dot{q}}_{2}\right)$ is expressed by Jacobian matrix **J**_**L**_ as

(5) $$\begin{array}{c} \left[\begin{array}{l} {\dot{L}}_{1}\\ {\dot{L}}_{2} \end{array}\right]=J_{L}\left[\begin{array}{l} {\dot{q}}_{1}\\ {\dot{q}}_{2} \end{array}\right]. \end{array}$$

### 3.2. Dynamic Modeling of PARM

**Figure 5:**
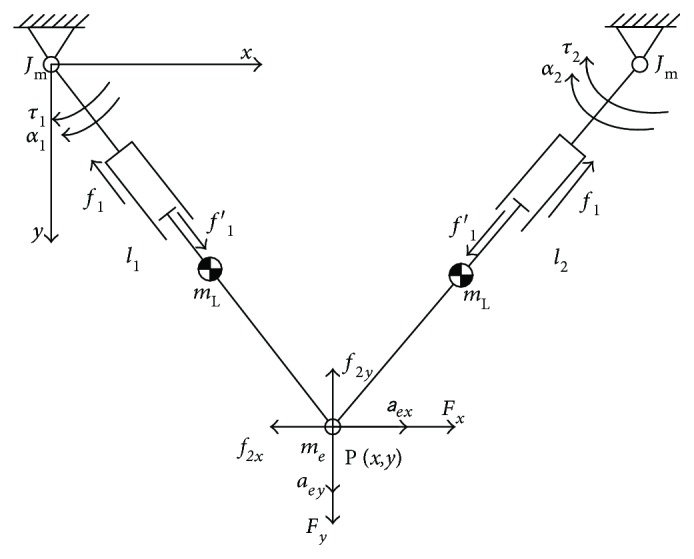
Robotic dynamics of the mechanism. In this diagram, *τ*_1_ and *τ*_2_ denote the motor torques; *f*_1_ and *f*_1_′ are the friction in the prismatic joint; *f*_2*x*_ and *f*_2*y*_ denote the friction between the end-effector and platform; *F*_*x*_ and *F*_*y*_ denote the interactive force between patients and the robot; *m*_L_, *m*_e_, and *J*_m_ denote the inertia of the link, end-effector, and the moment of inertia of the motor shaft, respectively; *α* and *a* represent the acceleration of the joints and end-effector, respectively.

**Figure 6:**
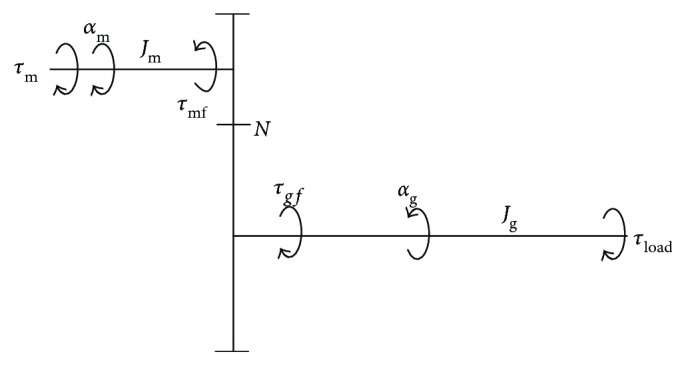
Dynamics of the motor-gear system. *J*_m_ and *J*_g_ denote the moments of inertia of the motor and gear shafts, respectively; *τ*_mf_, *τ*_gf_ and *α*_m_, *α*_g_ are the friction torques and angular accelerations of the two shafts, respectively; *τ*_m_ and *τ*_load_ represent the motor and load torques, respectively; *N* is the gear ratio.

For dexterous and accurate control of a manipulator, inertia and friction should be considered. In this study, it is hypothesized that three components constituted the motor torques. Namely, the first component counteracted friction; another component compensated the inertia of the end-effector, links, and motor-gear system; the last component generated the manipulator impedance and achieved the flexibility. The dynamic diagrams of the mechanism and motor-gear system are shown in Figures 5 and 6, respectively, where the arrows indicate the positive references.

#### 3.2.1. Friction Component

In this study, the principle of virtual work was utilized to deduce the equilibrium relations. Specifically, Δ*Q*(*δq*_1_, *δq*_2_) and Δ*P*(*δx*, *δy*) were virtual displacement of the motor joints and end-effector, respectively, and Δ*L*(*δL*_1_, *δL*_2_) was the corresponding virtual change of link length. In the patient-active training, the end-effector was mechanical impedance, and conversely, the human arm was regarded as mechanical admittance [29]. Thus, the equation can be written as

(6) $$\begin{array}{c} F\Delta P^{T}+{\tau_{f}}^{T}\Delta Q^{T}=f_{1}\mathrm{sign}\left(\Delta L\right)\Delta L^{T}-F_{2}\Delta P^{T}, \end{array}$$

where *τ*_f_ denotes the joint torques counteracting friction and external force; *F*(*F*_*x*_, *F*_*y*_) is the external force acting on the end-effector; *f*_1_ represents the friction in the prismatic joint; *F*_2_(*f*_2*x*_, *f*_2*y*_) means the friction between the end-effector and platform, and *f*_2_ = ‖*F*_2_‖. Based on (3) and (5), (6) can be deduced as

(7) $$\begin{array}{c} FJ_{p}\Delta Q^{T}+{\tau_{f}}^{T}\Delta Q^{T}=f_{1}\mathrm{sign}{\left(J_{L}\Delta Q^{T}\right)}^{T}J_{L}\Delta Q^{T}+f_{2}\frac{{\left(J_{p}\Delta Q^{T}\right)}^{T}}{||J_{p}\Delta Q^{T}||}J_{p}\Delta Q^{T}. \end{array}$$

To calculate the component of the joint torques which only counteracted the friction, the external force should be excluded. Eliminating the term Δ*Q*^*T*^, (7) can be written as

(8) $$\begin{array}{c} \tau_{f}=f_{1}{J_{L}}^{T}S_{1}+f_{2}{J_{p}}^{T}S_{2}, \end{array}$$

where the joint-parameter matrixes $S_{1}=\mathrm{sign}\left(J_{L}{\dot{Q}}^{T}\right)$ and $S_{2}=J_{p}{\dot{Q}}^{T}/\left\|J_{p}{\dot{Q}}^{T}\right\|$.

According to the motor-gear system shown in Figure 6, motor torque *τ*_1_ counteracting the friction is derived as

(9) $$\begin{array}{c} \tau_{1}=\frac{1}{N}\left(\tau_{f}+\mathrm{diag}\left(\mathrm{sign}\dot{Q}\right)\tau_{gf}\right)+\mathrm{diag}\left(\mathrm{sign}\dot{Q}\right)\tau_{mf}, \end{array}$$

where *τ*_mf_ and *τ*_gf_ denote the friction torques of motor and gear shafts, respectively, and *N* is the gear ratio.

#### 3.2.2. Inertia Component

The joint torque *τ*_*a*_ was assumed to compensate the inertia force generated by joint angular acceleration $\ddot{Q}\left({\ddot{q}}_{1},{\ddot{q}}_{2}\right)$ and end-effector acceleration $\ddot{P}\left(\ddot{x},\ddot{y}\right)$, which can be calculated as

(10) $$\begin{array}{c} \tau_{a}=\mathrm{diag}\left(J_{L},J_{R}\right){\ddot{Q}}^{T}+m_{e}J_{p}^{T}{\ddot{P}}^{T}, \end{array}$$

where *m*_e_ denotes the mass of the end-effector, and *J*_L_ and *J*_R_ are the moments of inertia of left and right links, respectively.

The end-effector acceleration could be obtained by the derivative of (3), which is given by

(11) $$\begin{array}{c} {\ddot{P}}^{T}={\dot{J}}_{p}{\dot{Q}}^{T}+J_{p}{\ddot{Q}}^{T}. \end{array}$$

In summary, the motor torque *τ*_2_ counteracting the inertia force could be calculated as

(12) $$\begin{array}{c} \tau_{2}=\frac{1}{N}\tau_{a}+\left(J_{m}+\frac{1}{N^{2}}J_{g}\right){\ddot{Q}}^{T}, \end{array}$$

where *J*_m_ and *J*_g_ are the moments of inertia of the motor and gear shafts, respectively.

## 4. Impedance Control for Assist-as-Needed Training

In impedance control, the end-effector behaves as a damped spring-mass system, which is represented in a single DoF system as

(13) $$\begin{array}{c} F_{\text{ext}}=M\ddot{x}+C\dot{x}+K\left(x-x_{d}\right), \end{array}$$

where *F*_ext_ denotes the external force; parameters *M*, *C*, and *K* are the dynamic parameters of the end-effector corresponding to mass, damping, and spring, respectively; *x*_*d*_ represents the desired equilibrium position, while *x* denotes the actual end-effector position.

**Figure 7:**
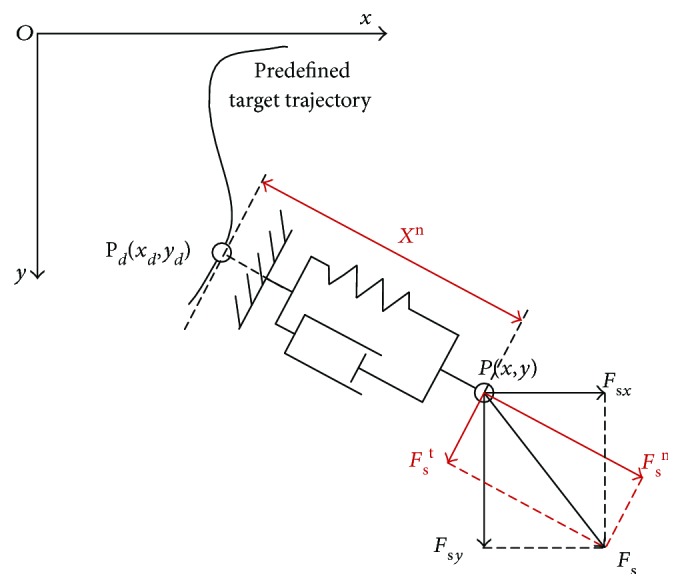
Diagram of the strategy based on the orthogonal deviation.

In robot-aided training, the predefined target trajectory *g*(*x*, *y*) meant the movement that patients were expected to track, which was, however, supposed to be different with the actual trajectory due to movement error. Actual trajectories were obtained by joint sensors and forward kinematics. Assume *P*_*d*_(*x*_*d*_, *y*_*d*_) denoted the desired position on the predefined target trajectory, when *P*(*x*, *y*) was the actual end-effector position. Since the reference position *P*_*d*_ determined the direction and magnitude of the impedance force, it was significant to search the appropriate reference position. In assist-as-needed training, patients determined the manipulator in terms of position, velocity, and acceleration; thus, the reference positions were variable with movements and associated with the real-time deviations. In this study, the strategy based on the orthogonal deviation was proposed to define the desired equilibrium positions for arbitrary predefined trajectories. As illustrated in Figure 7, the curve represents the predefined target trajectory, and force *F*_s*x*_ and *F*_s*y*_ are the interactive force detected by the three-axis force sensor in *x* and *y* directions, respectively. In this strategy, the direction of the actual position relative to the reference point was orthogonal to the tangent of the predefined trajectory at the equilibrium position, which indicated that the reference position *P*_*d*_ was the point on the predefined trajectory closest to the current position *P*. Therefore, the tangent component of the external force with respect to the equilibrium point provided the propulsive force *F*_s_^t^ along the trajectory, while the normal component force *F*_s_^n^ was supposed to be the impedance force (*F*_ext_) shown in (13), and *X*^n^ denotes the deviation input to the impedance control.

The dynamic modeling was used to calculate the motor torques to generate the required impedance according to deviations and impedance parameters. However, even though the dynamic modeling incorporated friction and inertia, control errors inevitably occurred in experiments; thus, the force sensor was utilized to obtain actual interactive force as feedback to reduce the errors. Since the acceleration and friction components have been discussed in Section 3, let *τ*_3_ be the motor torque-generating manipulator impedance, which is given by

(14) $$\begin{array}{c} \tau_{3}=\frac{1}{N}{J_{p}}^{T}\left[M\left(1+k\right){\ddot{X}}^{n}+C\left(1+k\right){\dot{X}}^{n}+K\left(1+k\right)X^{n}-kF_{s}^{n}\right], \end{array}$$

where *k* denotes the error feedback coefficient.

As the tangent force illustrated in Figure 7, for propulsion/resistance control, motor torque *τ*_t_ is implemented as

(15) $$\begin{array}{c} \tau_{t}=-\frac{1}{N}{J_{p}}^{T}F^{t}, \end{array}$$

where *F*^t^ denotes corresponding assistive/resistive tangent force along the predefined trajectory.

**Figure 8:**
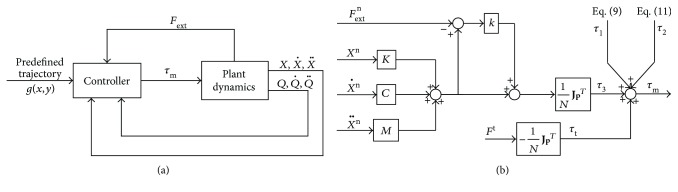
Control schemes of impedance and propulsion/resistance control. (a) Overview. (b) Controller diagram. *X*^n^, ${\dot{X}}^{n}$, ${\ddot{X}}^{n}$, and *F*_ext_^n^ are calculated by the strategy based on orthogonal deviation (Figure 7), and *F*^t^ denotes propulsion/resistance force.

Summarizing (9), (12), (14), and (15), as the control scheme shown in Figure 8, the motor torque for impedance control is calculated as

(16) $$\begin{array}{c} \tau_{m}=\tau_{1}+\tau_{2}+\tau_{3}+\tau_{t}. \end{array}$$

## 5. Simulations and Experiments

### 5.1. Impedance Parameter Determination

**Figure 9:**
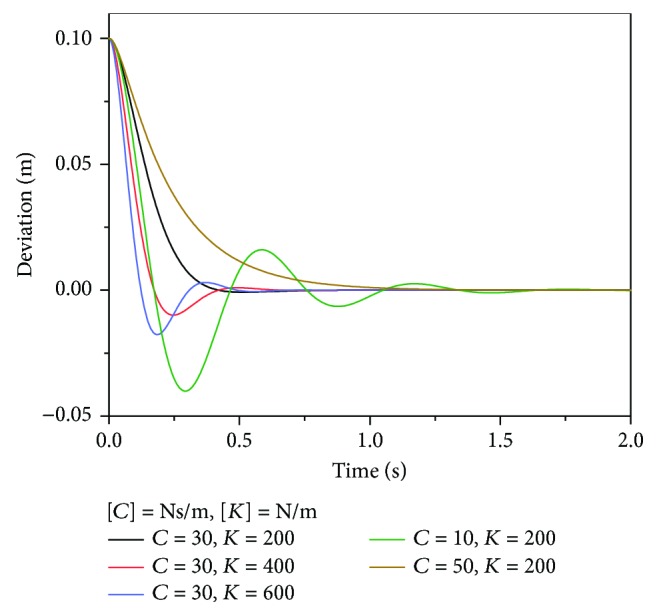
Manipulator responses with different impedance parameters.

Impedance parameters *M*, *C*, and *K*, which determined the dynamic behavior of the manipulator, were optimized by simulations. Specifically, it was supposed that the end-effector was released from the initial coordinates *P*_0_(0.5,0.4) m, while the equilibrium position was *P*_*d*_(0.4,0.4) m. *M* was set to 0.8 kg, which was the approximately actual mass of the end-effector, whereas the damping and stiffness coefficients *C* and *K* ranged from 10 to 50 Ns/m and 200 to 600 N/m, respectively. The dynamic responses of the manipulator in the absence of external interaction are shown in Figure 9. The result showed that the oscillation deteriorated with larger *K* and smaller *C* and the response time and overshoot were the least when *K* = 200 N/m and *C* = 30 Ns/m. Therefore, *K* and *C* were set to 200 N/m and 30 Ns/m for experiments, respectively.

### 5.2. Comparison between Experimental and Desired Responses

**Figure 10:**
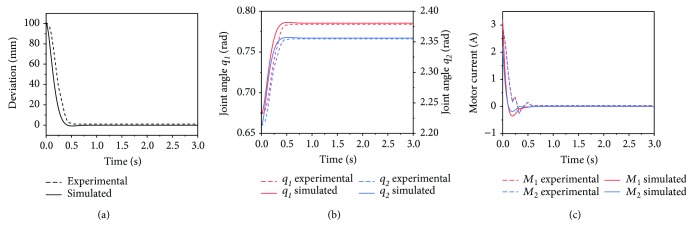
Experimental and desired results during the response from the deviated position to the equilibrium position with impedance control. (a) End-effector response. (b) Joint response. (c) Motor current response.

To validate the dynamic modeling and impedance control, an experiment of the deviation-regression response, which was the same as the simulation introduced in Section 5.1, was conducted. The comparisons between the experimental and desired results indicated by simulations are shown in Figure 10. As shown in Figures 10(a) and 10(b), the experimental responses of the end-effector and joints are approximately the same as the desired response, and the steady-state errors are approximately zero, indicating the accuracy and validity of the dynamic modeling and impedance control.  Figure 10(c) shows the simulated and experimental motor currents, where *M*_1_ and *M*_2_ denote the motor currents of motors 1 and 2, which actuate the *q*_1_ and *q*_2_, respectively. Consistent with Figures 10(a) and 10(b), the current responses also demonstrate the consistency between the actual dynamic performance and the modeling.

### 5.3. Movement Experiments

In order to discuss how impedance interacted with participants and the error pattern during movements, movement experiments were performed. A healthy male subject, who was 23 years old and left-handed, participated the experiments. The subject performed the movements of two representative types, that is, rectilinear repetitions and clockwise circular repetitions. Furthermore, each moment was performed under four conditions, that is, without impedance or resistance, with impedance only, with resistance only, and with impedance and resistance simultaneously. The resistance force was set to 8 N, which was implemented with (15), and the reference line in rectilinear repetition was set as *x*_*d*_ = 400 mm, while the radius of circular movements was 125 mm with respect to the center at *P*_c_(400, 425) mm. Since the movement speed could affect the accuracy, the repetition frequencies of rectilinear and circular movements were set to 0.5 Hz and 0.35 Hz, respectively. Each experiment lasted 100 seconds, and the interval time between two experiments was 1 hour to eliminate experimental interactions. The experimental protocol was approved by the Ethics Committee of Tsinghua University, Beijing, China.

**Figure 11:**
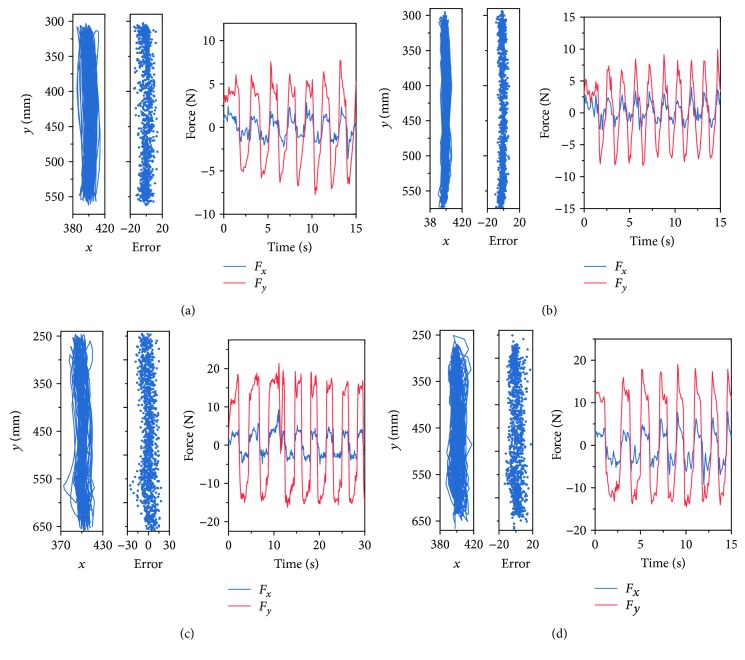
Trajectories, errors, and interactive force of the rectilinear movements (a) without impedance or resistance, (b) with impedance only, (c) with resistance only, and (d) with impedance and resistance simultaneously. *x* and error are measured by mm.

**Figure 12:**
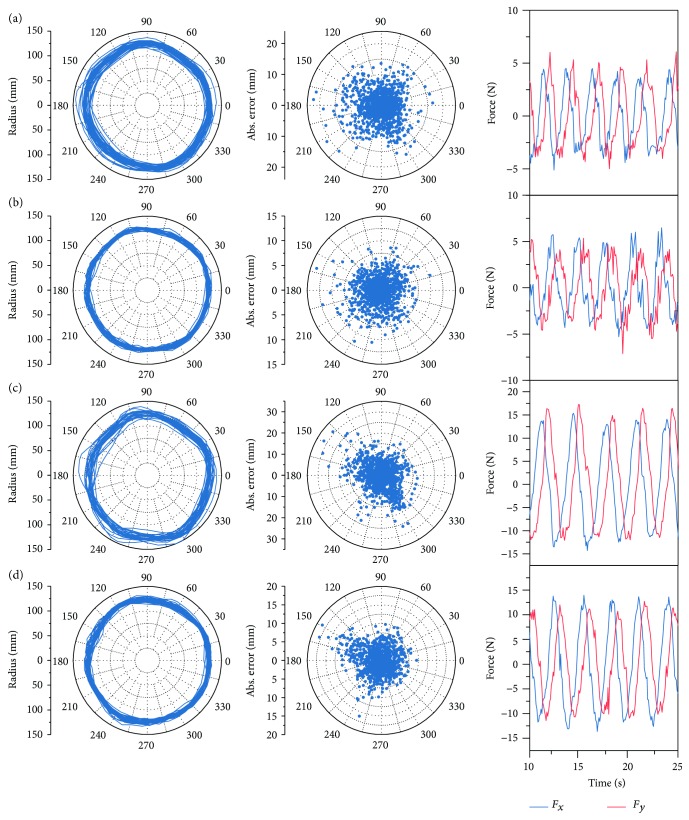
Trajectories, errors, and interactive force of the circular movements (a) without impedance or resistance, (b) with impedance only, (c) with resistance only, and (d) with impedance and resistance simultaneously.

The trajectories, errors, and interactive force of rectilinear and circular movements are represented in Figures 11 and 12, respectively. The repetitions in rectilinear movements were indicated by the alternation of *F*_*y*_ (Figure 11), while the force alternations in *x* and *y* directions both suggested the repetitions of circular movements (Figure 12). In circular movements, the signals of *F*_*x*_ and *F*_*y*_ were both sinusoidal with time, and the phrase of *F*_*y*_ lagged behind that of *F*_*x*_ by *π*/2. The maximal *F*_*y*_ in rectilinear movements and the maximal *F*_*x*_ and *F*_*y*_ in circular moments with resistance were larger than those without resistance by 8 N in average, which also validated the force control. Additionally, the results indicated that fewer errors were observed in the presence of impedance, whereas the performance deteriorated in the presence of resistance.

**Figure 13:**
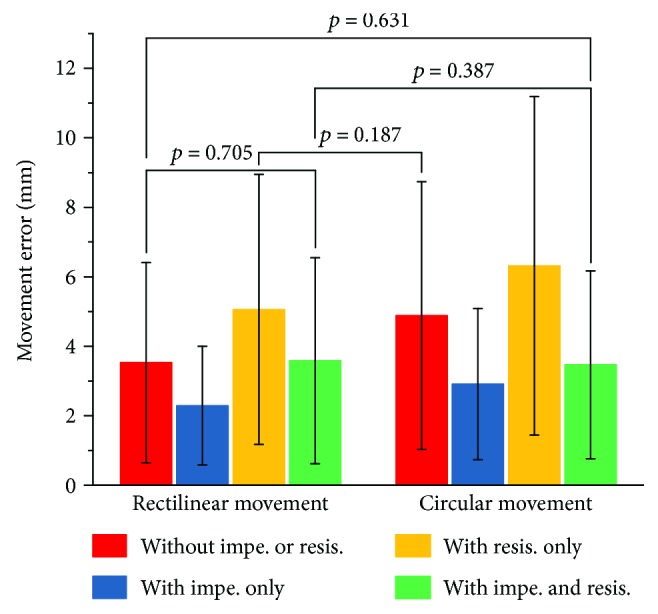
Statistics of absolute errors, where the brackets indicate nonsignificant differences (*p* > 0.05), and the significances at *p* < 0.001 are observed between other groups.

To assess the movement accuracy, the mean absolute error (MAE) and standard deviation (SD) were employed to evaluate the deviation of the movements. The brackets indicated the nonsignificant differences (*p* > 0.05), while the significances at *p* < 0.001 were observed between other groups (Figure 13). The results showed that the rectilinear movement with impedance only had minimal MAE and SD. Specifically, the impedance could significantly decrease the MAEs, whereas the MAEs were significantly increased in the presence of resistance, for both rectilinear and circular movements. Significant differences were also noted between the two groups under the same condition. In addition, the results suggested that the SDs were larger in the absence of impedance and in the presence of resistance, demonstrating that the impedance and resistance mediated the movements by affecting the MAE and SD simultaneously.

**Figure 14:**
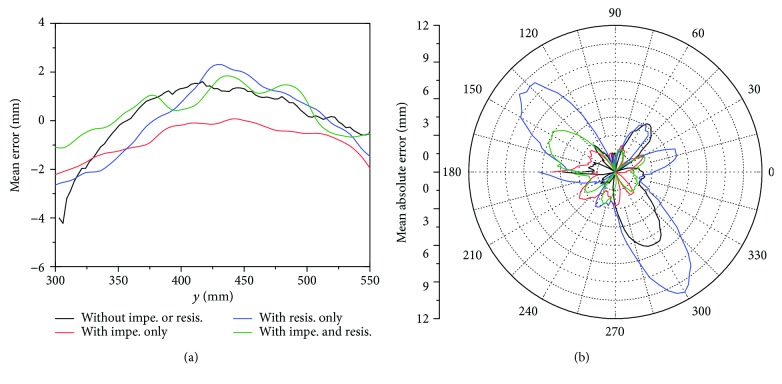
Statistic error patterns of the rectilinear and circular movements. (a) Mean errors with respect to *y*-position of rectilinear movements. (b) Mean absolute errors with respect to the polar angles of circular movements.

Since the impedance control could reduce the deviations significantly, it was essential to discuss the functional mechanism of the impedance for motor control, which incorporated two sides: theoretical and practical aspects. Theoretically, according to the impedance control proposed by Hogan [29], the main function of the impedance is to determine the interactive force given a deviation, in which case the manipulator is an impedance whereas the environment is an admittance. The generated impedance force is opposite to the deviation from the desired trajectory, which pulls the patient arm towards the desired trajectory as a “virtual damped spring” and alleviates movement deviations as compensative assistance. On the other hand, to interpret the functional mechanism of the impedance in practice, it is imperative to elucidate how the impedance interacts with participants, which is directly reflected by the error patterns in these movements. The kinematic analysis is shown in Figure 14, which indicates the statistic error patterns with respect to the positions. For rectilinear repetitions in *y* direction, the mean errors with respect to *y* positions presented the “arched deviations” within 300 to 550 mm, which reached the maximum at 425 mm (Figure 14(a)). The “arched effect” probably attributed to the inadequate synergy between the shoulder and elbow joints. Particularly, the contribution of the elbow joint motion was more than that of the shoulder joint; thus, the movements tended to present arc trajectories with respect to the elbow joint. As presented in Figure 14(a), the “arched effect” could be alleviated by impedance control. In circular repetitions, the maximal deviation occurred at polar angles of approximately 140 and 300 degrees (Figure 14(b)), which were close to the occasions when the elbow angles reached the maximum and minimum, respectively. Motor performance tended to decrease when close to the joint boundary, and the inadequacy of the elbow angles was supposed to be compensated by shoulder abduction and adduction, which might lead to movement errors but could be alleviated by impedance control. In summary, impedance environment was capable of alleviating movement deviations by compensating the synergetic inadequacy between shoulder and elbow joints, particularly when the movements were close to the joint boundary. Although the efficiency of impedance control regarding dynamic modeling and movement performance was demonstrated by experiments, nevertheless, the physical interaction between robot and human and the contribution to rehabilitation process still required further experiments performed on stroke patients.

## 6. Conclusions

This paper presented the design, dynamics, impedance control, and experiments of PARM: a parallel rehabilitation robot using impedance control to enhance interactive training. The parallel mechanism was introduced to reduce the inertia and improve the stiffness, capability, and precision. The motion perception and interaction could be improved by embedding an isometric screen. Apart from the mechanical design, the principle of virtual work and derivative of Jacobian matrix were incorporated to eliminate the frictional and inertial influence. Besides, the strategy based on orthogonal deviation was proposed to achieve the impedance control in assist-as-needed training. Comparisons between desired and experimental responses validated the dynamic modeling and impedance control. To investigate the influence of impedance for movements, movement experiments were also performed. The results showed that the errors of circular movements were mostly larger than those of rectilinear movements and demonstrated the significant differences between movements with/without impedance and resistance (*p* < 0.001), where the lowest and highest MAEs were noted in the presence of impedance and resistance, respectively. Furthermore, the “arched effect” was observed in rectilinear repetitions, and the deviation tended to occur when the motion was close to the joint boundary, but the impedance environment was capable of alleviating movement deviations by compensating the synergetic inadequacy between the shoulder and elbow joints. For the prospect of robot-assisted therapy, PARM could provide a reference for human-robot interaction in aspects of mechanical design, dynamic modeling, and assist-as-needed control.
